# Burnout and Engagement: Personality Profiles in Nursing Professionals

**DOI:** 10.3390/jcm8030286

**Published:** 2019-02-27

**Authors:** María del Carmen Pérez-Fuentes, María del Mar Molero Jurado, África Martos Martínez, José Jesús Gázquez Linares

**Affiliations:** 1 Department of Psychology, Faculty of Psychology, University of Almería, 04120 Almería, Spain; mmj130@ual.es (M.d.M.M.J.); amm521@ual.es (Á.M.M.); jlinares@ual.es (J.J.G.L.); 2 Department of Psychology, Universidad Autónoma de Chile, Región Metropolitana, Providencia 7500000, Chile

**Keywords:** personality, burnout, engagement, Big Five, healthcare personnel

## Abstract

The burnout syndrome, which affects many healthcare workers, has recently attracted wide interest due to the severe repercussions related to its effects. Although job factors determine its development, not all individuals exposed to the same work conditions show burnout, which demonstrates the importance of individual variables, such as personality. The purpose of this study was to determine the personality characteristics of a sample of nursing professionals based on the Big Five model. After having determined the personality profiles, we aimed to analyze the differences in burnout and engagement based on those profiles. The sample was made up of 1236 nurses. An ad hoc questionnaire was prepared to collect the sociodemographic data and the Brief Burnout Questionnaire, the Utrecht Work Engagement Scale and the Big Five Inventory-10 were used. The results showed that the existence of burnout in this group of workers is associated negatively with extraversion, agreeableness, conscientiousness and openness to experience, and it is associated positively with the neuroticism personality trait. These personality factors showed the opposite patterns with regard to engagement. Three different personality profiles were also found in nursing personnel, in which professionals who had a profile marked by strong neuroticism and low scores on the rest of the personality traits were the most affected by burnout.

## 1. Introduction

### 1.1. Burnout Syndrome and Individual Factors in Healthcare Professionals

The burnout syndrome, which mainly affects professionals who work directly with the beneficiaries of their services [1], is nowadays one of the most studied subjects in health and occupational psychology due to the results that have confirmed its severe economic and social consequences [2] to the health of workers and organizations [3,4].

Although job related factors are key determinants in developing burnout [5], not all individuals who experience the same working conditions develop the syndrome, which suggests the importance of individual factors in determining its appearance [6,7,8]. A study of burnout that focuses on an individualized approach through different profiles would provide knowledge for the change in levels of exhaustion and job commitment of workers or job engagement [9,10]. Along this line, hypotheses have been formulated, which show that burnout occurs in response to stress factors in the workplace. However, it is not the factors themselves that cause this exhaustion but the way that individuals face and manage them [11].

The personality characteristics of each individual play a relevant role in the way they work and the vitality and efficacy with which they perform their job [12,13]. They are also related to the perception of work events as stressful, such that individuals prone to anxiety are more vulnerable to job stress [14,15]. Studies suggest that personality factors are related to the occurrence of burnout syndrome in health professionals [16]. 

### 1.2. Burnout, Engagement and Personality in Nursing Personnel

There is significant debate in the literature on the theoretical construction of engagement and burnout. Some studies show that the engagement construct is the opposite of burnout [17,18,19]. Other studies have found that they are different concepts [20,21]. 

Consistent with this perspective, Maricuţoiu et al. [22] mention the existence of slight crossed effects between the variables, while others have found that the relationship between burnout and engagement is weak in healthcare personnel [23]. Engagement and burnout have also been found to have different patterns in relation to different variables. Thus, to explain burnout, it is essential to know both contextual and individual aspects, while engagement would be mainly determined by the latter [24,25] and more specifically, by the individual’s personality [26]. Thus, while the presence of burnout is associated with high neuroticism and low agreeableness, the engagement of healthcare professionals shows a strong association with the extraversion and conscientiousness personality factors. Therefore, even healthcare professionals with high scores in engagement can still suffer from burnout [27]. 

### 1.3. The Big Five Model and Its Association with Burnout in Healthcare Personnel 

One of the personality models that are most studied with regard to worker wellbeing is the Big Five [28]. Studies done on personality in specifically healthcare personnel have identified neuroticism as a factor, which has a strong association with burnout [16,18,29,30]. Individuals with high neuroticism levels are more prone to feeling angry, anxious, depressed or stressed [31,32]; are less able to control their emotions when faced with negative or stressful situations [30]; and exhibit immature defense mechanisms that increase their exhaustion [33]. In this vein, Iorga et al. [34] found that neuroticism, along with the difficulty in identifying their feelings, were the two variables that best explained the burnout scores of healthcare professionals.

Continuing with the Big Five model, conscientiousness has been associated with a lower score in burnout among nursing personnel. In turn, the locus of control acts as a moderating factor in this relationship, such that the force of the negative relationship between conscientiousness and burnout syndrome is lessened in those nursing professionals who have an internal locus of control [35]. Both conscientiousness and perfectionism are personality factors that mostly lead to a strong preoccupation for achieving results, which leads these individuals to deploy perseverant strict rules [36]. Both an exaggerated sense of conscientiousness and perfectionism have been described as common traits in healthcare professionals [37]. Perfectionism has been defined as the personality characteristic that refers to the struggle for correction and setting excessively high standards of performance, which can subsequently cause self-evaluation to be too critical [38]. Even just in common tasks, perfectionist individuals are not usually completely satisfied with the results, so exhaustion in these professionals is not only due to the task itself, but how they relate to it [34]. Within the study of personality and burnout, differences have been found in this syndrome based on the presence of maladaptive or healthy perfectionist personality traits [28]. People who have high maladaptive perfectionism tend to select coping strategies that are focused on emotions, which has been associated with the presence of burnout in healthcare workers [39]. In contrast, healthy perfectionism, which refers to employees who make an effort to reach these standards through initiative and motivation, show more innovation, which leads to lessening burnout [28]. 

Narcissism, which in the occupational sphere refers to persons who show a strong need to make their achievements visible and be recognized [40], is another personality factor that has been identified as a risk for developing burnout and more especially, with the dimensions of emotional exhaustion and depersonalization [41]. Personality traits pertaining to the Big Five model, such as agreeableness in interpersonal interaction, emotional stability, extraversion and openness to experience, have also been shown to be inversely related to the presence of burnout in healthcare professionals [29,33,42,43,44]. 

### 1.4. Personality Patterns and Their Relationship with the Burnout Syndrome in Nursing Professionals

Personality patterns constitute a network of stable traits, which are related to an individual’s behavior [45]. In the study of personality, Type D personality or distressed personality has been defined as the tendency to experience a high level of negative affectivity and social inhibition [46]. Distressed personality has been associated with burnout in workers [47]. Thus, healthcare professionals who have a Type D personality show more job stress and lower levels of professional satisfaction, which leads them to experience more burnout [48,49]. 

Another personality pattern that has been related to the presence of burnout in healthcare professionals is Type A profile, which refers to impulsive, competitive, impatient and aggressive individuals who have problems fighting job stress [50]. Healthcare professionals who show this behavior pattern have higher levels of job anxiety and emotional exhaustion [51,52]. While other studies, such as the one by Wlodarczyk and Pawliszewska [53] show that not all the components of Type A personality act in the same way. Thus, while aggressiveness acts as a predictor of burnout and job dissatisfaction, the factors of domination and effort to achieve have protective effects against this syndrome.

With regard to the types of personality, the review by Kennedy, Curtis, and Waters [54] mentions the possibility of certain factors being associated with the choice of training in nursing and with levels of stress, satisfaction and burnout. In this light, Jaracz et al. [55] suggested that most healthcare professionals show an anxious emotional temperament, which is characterized by the need to care for themselves and those around them. This temperament makes them especially vulnerable to anxiety, stress and burnout.

According to Zaninotto et al. [44], the complex interaction of burnout and personality traits is still not known in depth and the relationship between the sociodemographic, work and personality factors in nursing personal must be known in order to understand the presence of burnout [56]. Therefore, the objective of this study was to determine the Big Five personality characteristics in a sample of nursing professionals. In addition, having determined the personality profiles, we wanted to analyze the burnout and engagement scores based on those profiles.

## 2. Experimental Section

### 2.1. Participants

The sample was made up of 1236 nurses, of whom 85.5% (*n* = 1044) were women and the remaining 15.5% (*n* = 192) were men.

Participants were aged 21 to 57 years old with a mean age of 31.50 years (*SD* = 6.18). The mean age was 31.65 years for women (*SD* = 6.23) and 30.71 years for men (*SD* = 6.17). 

As for the areas they were working in, 32% (*n* = 396) were staff nurses and 21.9% (*n* = 271) were emergency staff, 11.4% (*n* = 141) performed their duties in the Intensive Care Unit, 10.7% (*n* = 132) in surgery, 2.3% (*n* = 28) said they worked in outpatient care and 4% (*n* = 50) worked in mental health. The remaining 17.6% (*n* = 218) were working in other areas.

Concerning their employment situation at the time of the survey, 69.3% (*n* = 857) were working with temporary contracts and 30.7% (*n* = 379) had permanent contracts. 

### 2.2. Instruments

We used an ad hoc sociodemographic questionnaire. This was a questionnaire prepared by the authors for the sociodemographic and employment variables in order acquire personal and job information and also determine the employment situation or work area. 

The Brief Burnout Questionnaire (CBB) [57] was used for evaluating this syndrome in the professionals. This instrument consists of 21 items grouped in three blocks corresponding to the background, elements and consequences of burnout. Thus, even though the objective of the questionnaire is the general evaluation of the professional burnout process, it is also concerned with the factors proposed in the Maslach and Jackson [58] model and components that precede and follow it. The response mode to the items is based on a Likert scale of 5 points. The response options change according to the content of the item (e.g., from “strongly disagree” to “strongly agree”, “never” to “always” and so on).The instrument’s reliability for the study sample was 0.87.

The Utrecht Work Engagement Scale (UWES) [59], specifically the version adapted to Spanish by Valdez and Ron [60], was used to evaluate engagement. This scale is made up of 17 items, which are answered on a seven-point Likert-type scale where 0 is “never” and 6 is “always”. These items are distributed into three factors: vigor (with regard to the energy with which the employee confronts his/her job), dedication (that is, the perception that the job performed makes sense) and absorption (related to the worker’s immersion in his/her work). The reliability of each of these factors in this study was 0.84, 0.90 and 0.81, respectively.

The Big Five Inventory-10 (BFI-10) [61] was used to evaluate personality traits. This questionnaire is a brief version of the original Big Five Inventory, which has 44 items (BFI-44) [62], and has been shown to have adequate psychometric properties despite its brevity. It contains five subscales (extraversion, agreeableness, conscientiousness, neuroticism and openness to experience), which are evaluated by two items. For this, the participants must answer on a Likert-type scale where 1 is “totally disagree” and 5 “totally agree”. The reliability analysis for each of the subscales showed a Cronbach’s alpha of 0.62 for extraversion, 0.67 for agreeableness, 0.71 for conscientiousness, 0.63 for neuroticism and 0.65 for openness to experience.

### 2.3. Procedure

This study was approved by the Bioethics Committee of the University of Almeria (Ref: UALBIO2017/011). Participation in the study was voluntary and participants were informed at all times of the purpose and the anonymity of their answers. 

The questionnaires were implemented on a Web platform and filled in individually online. In order to control any incongruent or random answers by participants, control questions were included. Any such cases were discarded from the study sample. 

### 2.4. Data Analysis

The descriptive statistics were analyzed for burnout and engagement and the personality factors. In addition, bivariate correlation analyses were performed to explore the relationship between variables. 

A two-step cluster analysis was carried out to establish the professional groups by personality factors. When the groups or clusters had been identified, the comparison of means (univariate analysis and multivariate analysis of variance) was performed to determine the existence of significant differences between the groups with respect to the burnout syndrome and engagement components. To determine which means were significantly different, the Scheffé post hoc comparison test was applied. 

Finally, a two-step cluster analysis was conducted on the group of professionals in the sample affected by burnout to determine their different personality profiles. 

The SPSS statistical package version 23 (SPSS Inc., Chicago, IL, USA) for Windows was used for data analysis and processing. 

## 3. Results

### 3.1. Personality, Burnout and Engagement in Nursing Professionals

As observed in Table 1, burnout correlated negatively with the three dimensions of engagement (Vigor: *r* = −39, *p* < 0.001; Dedication: *r* = −0.50, *p* < 0.001; Absorption: *r* = −0.28, *p* < 0.001) and correlated negatively with most of the personality factors (Extraversion: *r* = −0.14, *p* < 0.001; Agreeableness: *r* = −0.15, *p* < 0.001; Conscientiousness: *r* = −0.20, *p* < 0.001; Openness to experience: *r* = −0.18) and positively with Neuroticism (*r* = 0.20, *p* < 0.001).

In the relationships between engagement dimensions and personality factors, Vigor is observed to have a negative correlation with Neuroticism (*r* = −0.20, *p* < 0.001) and positive correlations with the other factors (Extraversion: *r* = 0.15, *p* < 0.001; Agreeableness: *r* = 0.15, *p* < 0.001; Conscientiousness: *r* = 0.30, *p* < 0.001; Openness to experience: *r* = 0.20, *p* < 0.001). The Dedication dimension has a negative correlation with Neuroticism (*r* = −0.18, *p* < 0.001) while it correlates positively with: Extraversion (*r* = 0.10; *p* < 0.001), Agreeableness (*r* = 0.15, *p* < 0.001), Conscientiousness (*r* = 0.26, *p* < 0.001) and Openness to experience (*r* = 0.22, *p* < 0.001). Finally, Absorption is negatively correlated with Neuroticism (*r* = −0.05, *p* < 0.05) and positively correlated with: Extraversion (*r* = 0.18; *p* < 0.001), Agreeableness (*r* = 0.10, *p* < 0.001), Conscientiousness (*r* = 0.16, *p* < 0.001) and Openness to experience (*r* = 0.13, *p* < 0.001).

### 3.2. Personality Profiles of Nursing Professionals: Differences in Burnout and Engagement

**Figure 1:**
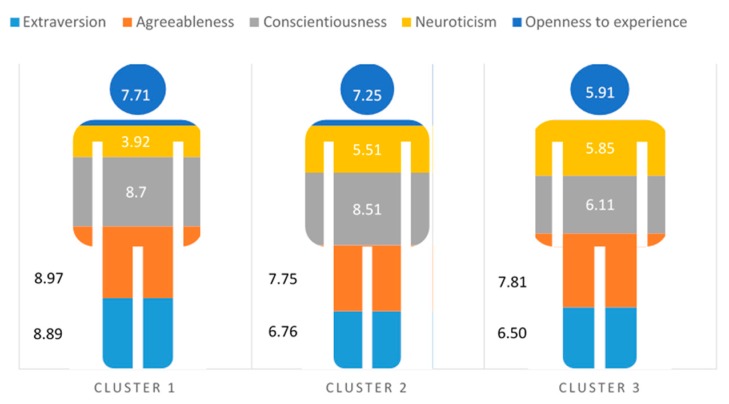
Mean scores on personality factors by cluster.

**Figure 2:**
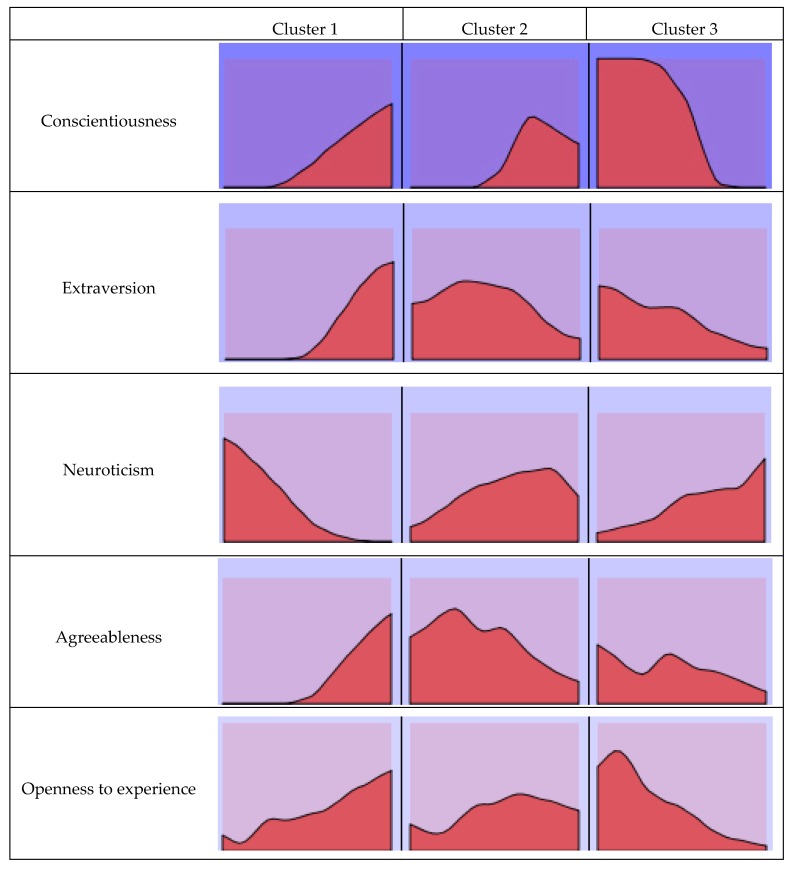
Cluster composition (*N* = 1236). The factors were organized in order of importance of input.

A two-step cluster analysis of the personality factors was performed to form the groups. Three groups resulted from the inclusion of these variables (Figure 1) with the following distribution: 39% (*n* = 482) of the participants in Cluster 1, 37.7% (*n* = 466) in Cluster 2 and 23.3% (*n* = 288) in Cluster 3. The following table summarizes the means of the personality factors for the total sample of participants and each of the clusters (Table 2 and Figure 2).

The first group resulting from the cluster analysis (Cluster 1) is characterized by scores above the mean of the total sample in Extraversion (M = 8.89), Agreeableness (*M* = 8.97), Conscientiousness (*M* = 8.70) and Openness to experience (*M* = 7.71), while Neuroticism had a mean below the total sample (*M* = 3.92).

The second group (Cluster 2) identifies professionals with scores above the mean of the total sample in Conscientiousness (*M* = 8.71), Neuroticism (*M* = 5.51) and Openness to experience (*M* = 7.25). In this case, for Extraversion (*M* = 6.76) and Agreeableness (*M* = 7.75), the scores were below the mean of the total sample. 

The third group (Cluster 3) clusters professionals with mean scores below the total sample in almost all the personality factors: Extraversion (*M* = 6.50), Agreeableness (*M* = 7.81), Conscientiousness (*M* = 6.11) and Openness to experience (*M* = 5.91), except Neuroticism (*M* = 5.85), for which the mean was above that for the total sample.

After classification into groups based on the three-cluster solution, a univariate analysis of variance (ANOVA) was performed for burnout and multivariate analysis (MANOVA) using the three dimensions of engagement. 

**Figure 3:**
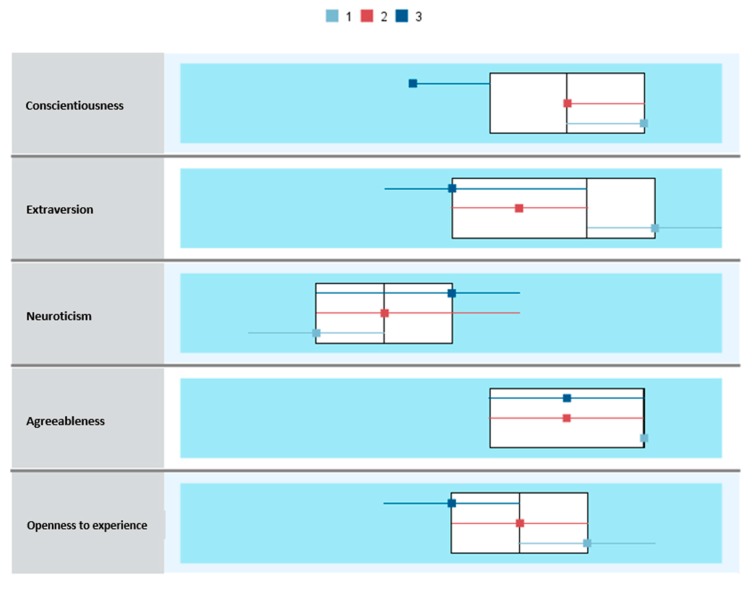
Comparison of clusters (*N* = 1236).

The results of the comparative analysis between profiles (Figure 3) by burnout syndrome are shown in Table 3, which demonstrated statistically significant differences between clusters (*F*_(2,1233)_ = 33.87, *p* < 0.001, *η_p_*^2^ = 0.05, observed power = 1.0).

The post hoc comparisons show that Cluster 3 (with scores below the mean in all the personality factors, except Neuroticism with a mean above that in the total sample) had a significantly higher score (*M* = 21.66) than the rest of the groups. In turn, the Cluster 2 score (*M* = 20.70) was significantly higher than Cluster 1 (*M* = 18.93).

A comparison of personality profiles with engagement dimensions were performed by the multivariate analysis of variance (MANOVA). 

Homogeneity of covariance was examined by the Box *M* test and the null hypothesis of data fit was rejected (*M_Box_* = 79.48, *F* = 6.59, *p* < 0.05). The multivariate comparison demonstrated the existence of significant between-group differences (Wilks’ Lambda = 0.88, *F*_(3, 1231)_ = 25.16, *p* < 0.001, *η_p_*^2^ = 0.05, observed power = 1.0). 

After analyzing this relationship individually for each of the dependent variables (Vigor, Dedication, Absorption), we found that the results were statistically significant in all cases (Table 4). 

In Vigor, there were significant differences between groups (*F*_(2,1233)_ = 65.37, *p* < 0.001, *η_p_*^2^ = 0.09, observed power = 1.0). Post hoc comparisons (Table 5) showed that Cluster 1 (with scores above the mean in all personality factors, except Neuroticism, which was below the total sample) had a significantly higher score (*M* = 28.95) than the rest of the groups. Furthermore, Cluster 2 (*M* = 26.87) had a significantly higher score than Cluster 3 (*M* = 24.50).

Significant differences were also found between the three clusters for the Dedication dimension (*F*_(2,1233)_ = 52.05, *p* < 0.001, *η_p_*^2^ = 0.07, observed power = 1.0). The post hoc comparisons showed that Cluster 1 (*M* = 25.30) had the highest mean score in Dedication, with statistically significant differences from Cluster 2 (*M* = 23.62) and the comparison between Cluster 2 and Cluster 3 (*M* = 21.65). 

Finally, significant between-groups differences were also found in the Absorption dimension of engagement (*F*_(2, 1233)_ = 19.46, *p* < 0.001, *η_p_*^2^ = 0.03, observed power = 1.0). The post hoc comparisons indicated that Cluster 1 had a significantly higher score (*M* = 25.55) than the rest of the groups. In turn, the score for Cluster 2 (*M* = 24.17) was significantly higher than Cluster 3 (*M* = 22.85).

### 3.3. Personality Profiles of Nursing Professionals with Burnout Syndrome

Selecting the part of the sample affected by the burnout syndrome, a two-step cluster analysis was conducted to test for different profiles by combining the personality factors analyzed. Two groups or clusters were found (from this point forward, Burnout Cluster): Burnout Cluster 1, which represented 46.6% (*n* = 102) of the subsample selected, and Burnout Cluster 2, where the remaining 53.4% (*n* = 117) were clustered.

**Figure 4:**
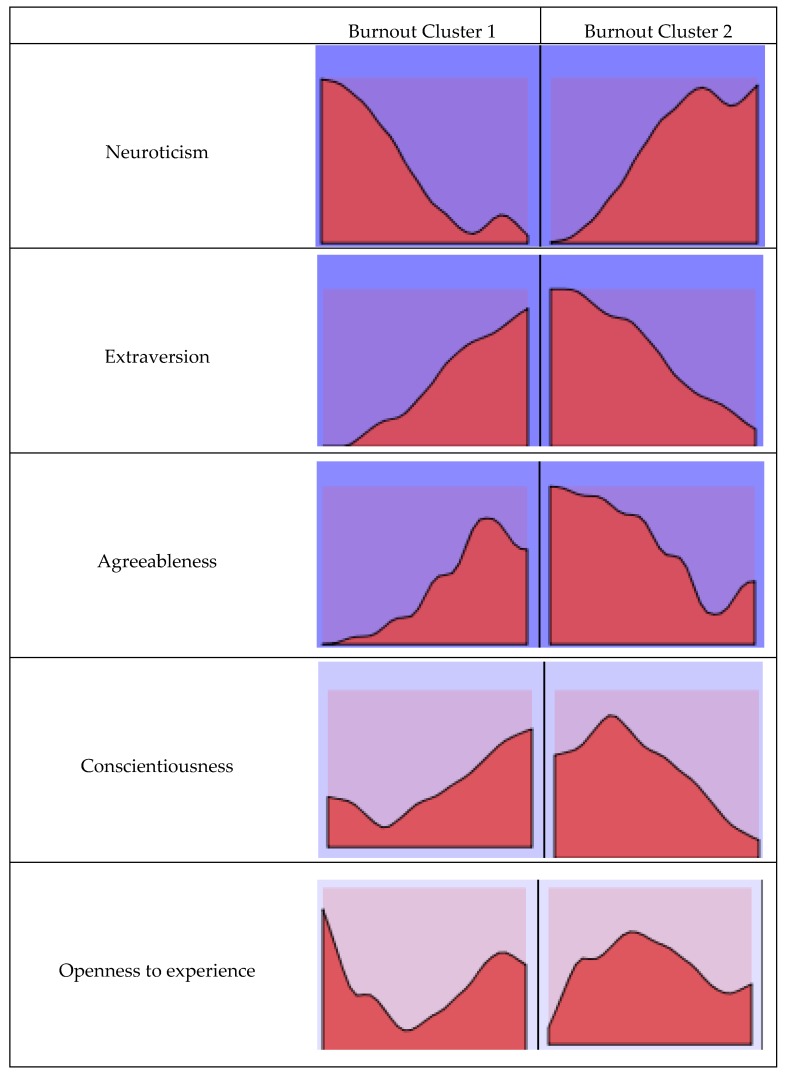
Cluster composition (*n* = 219). The factors were organized in order of importance of input.

Unlike the clusters resulting for the total sample of professionals, in this case (affected by burnout), Neuroticism appeared as the most relevant factor in determining the profiles (Figure 4). 

**Figure 5:**
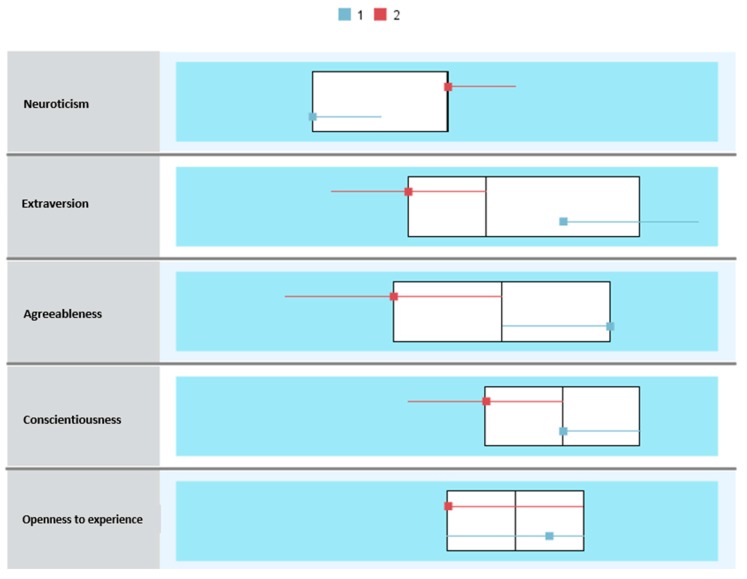
Comparison of clusters (*n* = 219).

Burnout Cluster 1 identifies professionals with scores above the mean in all the personality factors (Extraversion: *M* = 8.30, Agreeableness: *M* = 8.70, Conscientiousness: *M* = 8.09 and Openness to experience: *M* = 6.99), except Neuroticism (*M* = 4.47), which was below the mean in professionals affected by the syndrome. On the contrary, Burnout Cluster 2 grouped professionals with scores below the mean in almost all the personality factors (Extraversion: *M* = 6.08, Agreeableness: *M* = 7.28, Conscientiousness: *M* = 7.14 and Openness to experience: *M* = 6.76), while Neuroticism (*M* = 5.48) had a score above the subsample of professionals affected by burnout (Figure 5).

Finally, to check whether the differences in the degree of burnout between the two personality profiles of those affected by the syndrome were statistically significant, a Student’s *t*-test for independent samples was carried out. The results showed that the differences between the two profiles were not statistically significant (*t*_(217)_ = −1.54, *p* = 0.12). 

Nevertheless, when comparing means, a higher mean score was observed in burnout for Burnout Cluster 2 (*M* = 28.08, *SD* = 3.32) than Burnout Cluster 1 (*M* = 27.48, *SD* = 2.35).

## 4. Discussion

This study analyzed the relationship between certain personality factors and the presence of burnout in nursing personnel. The results showed that burnout in this group of workers was associated negatively with Extraversion, Agreeableness, Conscientiousness and Openness to experience, but had a positive relationship with Neuroticism. These results agree with the findings of other studies, where all personality factors based on the Big Five Model were shown to be negatively related to development of the burnout syndrome in healthcare workers [29,33,42,43,44], except neuroticism, which was positively related [16,18,29,30].

On the contrary, these personality factors showed the opposite pattern in relation to engagement in nursing professionals. Thus, while neuroticism showed a negative relationship in this construct, the Extraversion, Conscientiousness, Agreeableness and Openness to experience factors correlated positively. Although these results are insufficient to resolve the debate concerning burnout and engagement as different constructs or different poles of the same construct, it can be said that there are interrelated effects, which coincides with the results of other authors [22]. 

When the personality profiles in nursing personnel were analyzed, three different groups were found. The first one had positive scores on all the personality factors analyzed, except neuroticism. The second profile referred to professionals that were less agreeable and extroverted than the rest but showed high scores on the rest of the factors (namely conscientiousness, openness to experience and neuroticism). Finally, the third group from the cluster analysis was made up of professionals with a personality profile that was contrary to the one shown by the first group. Thus, the third group was characterized by showing scores above the mean in neuroticism and below in the rest. After comparing the burnout and engagement scores in these three profiles, the third group, which was marked by low scores on all the traits except neuroticism, was found to have the most burnout syndrome, followed by the second group made up of professionals with low agreeableness and extraversion and high neuroticism scores (although the scores on this factor were lower than in the third group). These results are consistent with those found by Iorga et al. [34], who identified neuroticism as the key personal element in developing burnout. The second group, which was also the second most burnt out, show a similar pattern to the one established in the literature as Type D personality, which was previously identified as increasing the risk of developing burnout [47,48,49].

Furthermore, the highest scores in engagement were shown by the first group, which was marked by high scores in Extraversion, Agreeableness, Openness to experience and Conscientiousness [18,30]. This is followed by the second and third groups, respectively.

Finally, several different personality profiles were found among the nursing personnel who were suffering from burnout. After identifying the professionals who had this syndrome, a cluster analysis was done, which indicated two different groups. The second, which was made up of workers with scores below the mean in all the traits in the Big Five model except neuroticism, showed higher burnout scores than the first group where the opposite personality pattern was found. Although these differences were not significantly higher, they highlight again the importance of the neuroticism factor in developing burnout and even in showing higher levels of this syndrome. 

## 5. Conclusions

Personality factors are relatively stable traits, which influence behavior and the way that healthcare professionals face daily situations. Certain factors, such as neuroticism, show a strong relationship with the development of burnout in nursing personnel. Knowing how the personality of the individual can affect the development of this ever more prevalent phenomenon is a challenge. At the same time, this provides an opportunity for optimizing human resources within the organization and improving the general quality of life of healthcare professionals. 

Among the limitations of the study is the selection of questionnaires. In the first place, the CBB [57] cannot find the influence of the personality variables on each of the burnout factors as it only provides a total score. On the other hand and according to the authors of the Big Five Inventory-10 [61], although the questionnaire’s properties are adequate, there are losses in evaluating personality adequately due to its brevity. Therefore, we recommend that future studies should use the 44-item version of the Big Five Inventory [62] and utilize other questionnaires that evaluate the various factors of the burnout syndrome more precisely. 

This study shows the need for continued analysis of the individual factors and more specifically, personality, in relation to the burnout syndrome. At the same time, other factors should also be analyzed. Thus, new studies focusing on the role of personality along with other factors, such as coping strategies or job stress levels, would be appropriate to demonstrate the decisive role of personality in developing burnout given exposure to the same work factors.

## Figures and Tables

**Table 1:** Personality, burnout and engagement. Bivariate correlations.

Variables	1	2	3	4	5	6	7	8	9
**1. Extraversion**	-								
**2. Agreeableness**	0.20 ***	-							
**3. Conscientiousness**	0.25 ***	0.19 ***	-						
**4. Neuroticism**	−0.24 ***	−0.19 ***	−0.26 ***	-					
**5. Openness to experience**	0.20 ***	0.10 ***	0.30 ***	−0.20 ***	-				
**6. Burnout syndrome**	−0.14 ***	−0.15 ***	−0.20 ***	0.20 ***	−0.18 ***	-			
**7. Vigor**	0.15 ***	0.15 ***	0.30 ***	−0.20 ***	0.20 ***	−0.39 ***	-		
**8. Dedication**	0.10 ***	0.15 ***	0.26 ***	−0.18 ***	0.22 ***	−0.50 ***	0.83 ***	-	
**9. Absorption**	0.18 ***	0.10 ***	0.16 ***	–0.05 *	0.13 ***	−0.28 ***	0.77 ***	0.74 ***	-

* *p* < 0.05, *** *p* < 0.001.

**Table 2:** Mean scores for the total sample and clusters (*N* = 1236).

Variables	Total Sample (*N* = 1236)	Cluster		
		1 (*n* = 482)	2 (*n* = 466)	3 (*n* = 288)
**Extraversion**	*M* = 7.53 (*SD* = 1.88)	*M* = 8.89 (*SD* = 1.00)	*M* = 6.76 (*SD* = 1.76)	*M* = 6.50 (*SD* = 1.87)
**Agreeableness**	*M* = 8.24 (*SD* = 1.23)	*M* = 8.97 (*SD* = 0.72)	*M* = 7.75 (*SD* = 1.26)	*M* = 7.81 (*SD* = 1.27)
**Conscientiousness**	*M* = 8.02 (*SD* = 1.39)	*M* = 8.70 (*SD* = 0.96)	*M* = 8.51 (*SD* = 0.77)	*M* = 6.11 (*SD* = 0.98)
**Neuroticism**	*M* = 4.97 (*SD* = 1.72)	*M* = 3.92 (*SD* = 1.16)	*M* = 5.51 (*SD* = 1.65)	*M* = 5.85 (*SD* = 1.74)
**Openness to experience**	*M* = 7.12 (*SD* = 1.76)	*M* = 7.71 (*SD* = 1.64)	*M* = 7.25 (*SD* = 1.55)	*M* = 5.91 (*SD* = 1.68)

**Table 3:** Burnout and personality profiles. Univariate analysis of variance and post hoc.

Variables	Cluster	*N*	*Mean*	*SD*	ANOVA		Difference in Means
					*F*	Sig.	
**Burnout**	**Cluster 1 (c1)**	482	18.93	4.56	33.87	0.000	|g1–g2|***|g2–g3|*|g1–g3|***
	**Cluster 2 (c2)**	466	20.70	4.79			
	**Cluster 3 (c3)**	288	21.66	4.84			

* *p* < 0.05, *** *p* < 0.001.

**Table 4:** Multivariate analysis (between-subject effects by cluster) based on the engagement dimensions.

Engagement	Cluster 1 (*n* = 482)		Cluster 2 (*n* = 466)		Cluster 3 (*n* = 288)					
	*M*	*SD*	*M*	*SD*	*M*	*SD*	*F*	*p*	*η_p_* ^2^	Observed power
**Vigor**	28.95	4.42	26.87	5.20	24.50	6.46	65.37	0.000	0.09	1.00
**Dedication**	25.30	4.08	23.62	4.75	21.65	5.96	52.05	0.000	0.07	1.00
**Absorption**	25.55	5.34	24.17	5.76	22.85	6.86	19.46	0.000	0.03	1.00

**Table 5:** Post hoc tests by cluster for engagement dimensions.

Engagement	Difference in Means		
	Cluster 1 vs. Cluster 2	Cluster 1 vs. Cluster 3	Cluster 2 vs. Cluster 3
**Vigor**	2.07 ***	4.44 ***	2.37 ***
**Dedication**	1.68 ***	3.65 ***	1.97 ***
**Absorption**	1.38 **	2.70 ***	1.32 **

** *p* < 0.01; *** *p* < 0.001.
